# Increased risk of obstructive sleep apnoea in women with polycystic ovary syndrome: a population-based cohort study

**DOI:** 10.1530/EJE-18-0693

**Published:** 2019-02-13

**Authors:** Balachandran Kumarendran, Dana Sumilo, Michael W O’Reilly, Konstantinos A Toulis, Krishna M Gokhale, Chandrika N Wijeyaratne, Arri Coomarasamy, Wiebke Arlt, Abd A Tahrani, Krishnarajah Nirantharakumar

**Affiliations:** 1 Institute of Applied Health Research, University of Birmingham, Birmingham, UK; 2 Department of Community and Family Medicine, Faculty of Medicine, University of Jaffna, Jaffna, Sri Lanka; 3 Institute of Metabolism and Systems Research, University of Birmingham; 4 Centre for Endocrinology, Diabetes and Metabolism, Birmingham Health Partners, Birmingham, UK; 5 Department of Obstetrics and Gynecology, Faculty of Medicine, University of Colombo, Colombo, Sri Lanka; 6 Health Data Research, Birmingham, UK

## Abstract

**Objective:**

Obesity is very common in patients with obstructive sleep apnoea (OSA) and polycystic ovary syndrome (PCOS). Longitudinal studies assessing OSA risk in PCOS and examining the role of obesity are lacking. Our objective was to assess the risk of OSA in women with vs without PCOS and to examine the role of obesity in the observed findings.

**Design:**

Population-based retrospective cohort study utilizing The Health Improvement Network (THIN), UK.

**Methods:**

76 978 women with PCOS and 143 077 age-, BMI- and location-matched women without PCOS between January 2000 and May 2017 were identified. Hazard ratio (HR) for OSA among women with and without PCOS were calculated after controlling for confounding variables using multivariate Cox models.

**Results:**

Median patient age was 30 (IQR: 25–35) years; median follow-up was 3.5 (IQR: 1.4–7.1) years. We found 298 OSA cases in PCOS women vs 222 in controls, with incidence rates for OSA of 8.1 and 3.3 per 10 000 person years, respectively. Women with PCOS were at increased risk of developing OSA (adjusted HR = 2.26, 95% CI: 1.89–2.69, *P* < 0.001), with similar HRs for normal weight, overweight and obese PCOS women.

**Conclusions:**

Women with PCOS are at increased risk of developing OSA compared to control women irrespective of obesity. Considering the significant metabolic morbidity associated with OSA, clinicians should have a low threshold to test for OSA in women with PCOS. Whether OSA treatment has an impact on PCOS symptoms and outcomes needs to be examined.

## Introduction

Polycystic ovary syndrome (PCOS) is the commonest endocrine disorder in women of reproductive age (prevalence 8–13%) (1). Chronic anovulation, hyperandrogenism and ovarian polycystic morphology are the defining features of PCOS.

PCOS is associated with multiple comorbidities including obesity, insulin resistance (IR), dyslipidaemia, gestational diabetes (GDM), type 2 diabetes (T2D), hypertension, non-alcoholic fatty liver disease, impaired quality of life (QOL), cardiovascular disease (CVD) and mortality amongst others (2, 3, 4). Weight loss (by lifestyle intervention or bariatric surgery) remains the only specific treatment for PCOS. The remaining treatments are mainly symptomatic (oral contraceptives for irregular periods, anti-androgens for hirsutism, ovulation induction for infertility) (5). Hence, there is a need for better understanding of the pathogenesis of PCOS-related metabolic risk and comorbidities in order to develop effective therapies (6).

Obstructive sleep apnoea (OSA) is common, affecting 17–26% of men and 9–28% of women; the prevalence being lower in women of reproductive age compared to men (7). OSA is characterized by instability in the upper airways during sleep, leading to recurrent upper airway obstructions, sleep architecture disruption and cyclical changes in heart rate, blood pressure, sympathetic activity, intrathoracic pressure and oxygen saturations (7, 8). Obesity is very common in patients with OSA and PCOS (9). In addition, OSA is associated with similar comorbidities to PCOS, such as IR, GDM, T2D, hypertension, impaired QOL, CVD and mortality (7, 10). Hence, it is plausible that OSA and PCOS might co-exist and that either condition could contribute to the comorbidities of the other (9). This is further supported by a recent systematic review that showed that OSA prevalence in women with PCOS was 32% (95% CI: 13–55%) (11). However, these studies were of small size (*n* < 60), at risk of selection bias and cross-sectional design, barring the determination of the direction of relationship. Additionally, the study populations exclusively comprised patients with grade II obesity or higher. Hence, there is a need to examine the relationship between OSA and PCOS in a longitudinal population-based study, which allows to assess the impact of obesity.

Several mechanisms, other than obesity and insulin resistance, might increase the likelihood of OSA in women with PCOS compared to women without PCOS of similar adiposity including hyperandrogenism, low progesterone (due to anovulation) and increased oxidative stress (9).

We hypothesized that women with PCOS are at increased risk of developing OSA compared to women without PCOS regardless of the degree of obesity. The primary aim of this study was to assess the risk of incident OSA in women with PCOS vs women without PCOS and examine the role of obesity in the observed relationships. A secondary aim was to assess predictors of incident OSA in women with PCOS.

## Subjects and methods

For full description of the methods please refer to the online supplement.

### Ethics statement

The use of THIN data for research was approved by the South-East Multicenter Research Ethics Committee in 2003 without the need for informed consent. As per requirement of the ethical approval, further registration and authorization for this project were obtained from the relevant Scientific Review Committee (17THIN026).

### Study design and setting

We conducted a matched-controlled retrospective cohort study using data from UK general practices contributing to The Health Improvement Network (THIN) electronic database, as we have in previous studies (2, 12, 13).

### Study population

The open cohort extended from the 1st January 2000 (study start date) to the 15th May 2017 (study end date).

*Inclusion criteria*: All women who were aged 18–50 years at the index date (study entry) and had a documentation of PCOS at any time during the study period were included in the exposed group. Patients with any documentation of OSA prior to the index date were excluded. Women without documented PCOS at any time during the study period were included in the unexposed (control) arm. The index date was defined as the date of first documentation of PCOS for newly diagnosed cases and from the date patient became eligible if the first documentation of PCOS was prior to the eligibility date (for existing cases) (Supplementary Fig. E1, see section on supplementary data given at the end of this article).

Each exposed patient was randomly matched to two unexposed patients (1:2 ratio) for general practice, age at index date and BMI (14, 15).

To minimize the immortal time bias, each randomly matched eligible unexposed patient was assigned the same index date as their corresponding exposed patient (16). Follow-up end date (exit date) was determined from the earliest occurrence of the first documentation of OSA, transfer to another practice, death or study end.

### Selection of Read Codes and PCOS definition

Read Codes to define PCOS, OSA and covariates were compiled using a methodical Read Code search strategy (see Supplementary Panel E1 and Table E1) Since there is a possibility of misclassification between PCOS and polycystic ovaries (PCO) due to the resemblance of codes during data entry, they have been combined in prevalence studies using general practice electronic databases (17).

### Statistical analysis

Potential confounders and covariates were chosen based on biological plausibility and links to the exposure and outcome of interest (PCOS and OSA respectively). These included age, Townsend social deprivation index (14), BMI, smoking status, diabetes mellitus, impaired glucose regulation (18), hypertension (19), hypothyroidism, antiandrogen medication and metformin. The critical value for statistical significance was set at 5% and 95% confidence intervals were used in the population estimate of hazard ratios. Further analysis included calculating the number and percentage of incident cases, person years and incidence rates. Unadjusted and adjusted hazard ratios were estimated using Cox regression models.

Sensitivity analysis was carried out to assess selection bias due to case definition (PCOS and PCO vs PCOS only) and survival bias due to inclusion of prevalent cases (who had the documentation of PCOS prior to becoming eligible for the study) (20). In the primary analysis, we did not consider ethnicity as a covariate due to high missing values (48.9%). In a sensitivity analysis we assigned a separate ethnicity category for missing values and included into the Cox model as an additional covariate.

Another model confined to PCOS cases (excluding controls) was used to assess the association of PCOS with OSA while considering PCOS phenotypes and antiandrogen medication as covariates. STATA MP version 14.2 was used for data cleaning and analysis (21).

## Results

### Study population characteristics

**Figure 1:**
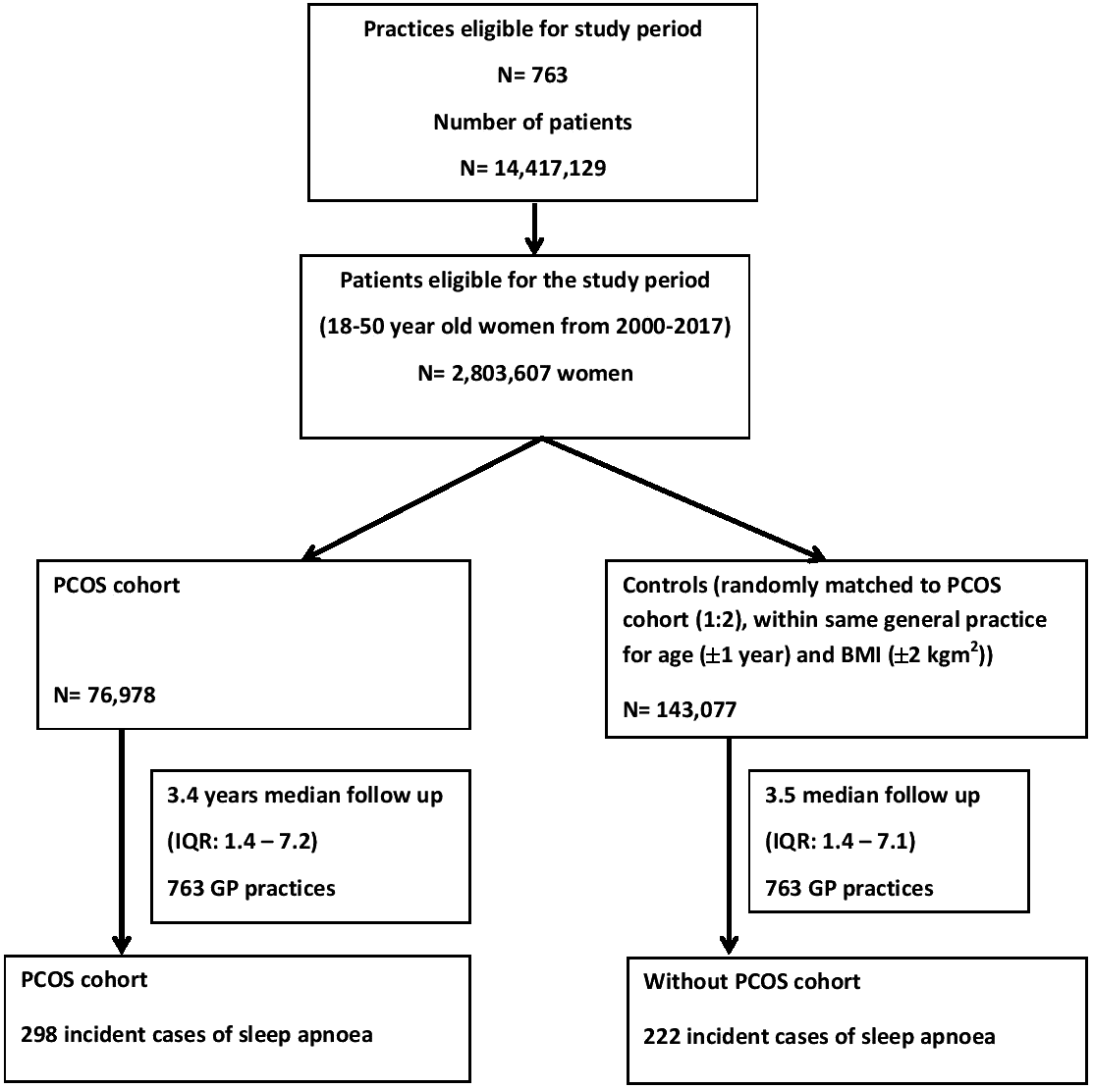
Selection of patients with polycystic ovary syndrome (PCOS) and controls. A total of 14 417 129 patients were registered with 763 eligible practices during 2000 to 2017. Of these, 2 803 607 women were of 18 to 50 years old and were eligible. According to the criteria indicated in ‘methods’ section, there were 76 978 women with PCOS in PCOS cohort. The control cohort had 143 077 women who were matched within same general practice for age and BMI at a ratio of 1:2. There were 298 and 222 incident cases of sleep apnoea in PCOS and control cohorts respectively.

The study population included 76 978 women with PCOS and 143 077 matched control women from 763 general practices registered with THIN (Fig. 1). The median follow-up was 3.5 years (IQR: 1.38 to 7.14) with no significant difference between women with and without PCOS (Fig. 1). There was no obvious difference between women with and without PCOS with regards to age (median 30 years, IQR: 25–35), Townsend index for social deprivation, BMI and smoking status (Table 1). When compared to controls, women with PCOS were more likely to have T2D (2.2 vs 1.0%), hypertension (3.0 vs 2.0%), hypothyroidism (3.9 vs 2.3%) and impaired glucose regulation (0.6% vs 0.3%) (Table 1). The PCOS group was also more likely to develop diabetes mellitus during the follow-up when compared to control group (5.6 vs 2.6%).

**Table 1:** Baseline characteristics of women with PCOS and control women without PCOS.

Characteristics	With PCOS (*n* = 76 978)	Without PCOS (*n* = 143 077)
Age (years; mean (s.d.))	30.2 (7.4)	30.4 (7.3)
Townsend score^*^		
1	13 865 (18.0%)	25 951 (18.1%)
2	12 716 (16.5%)	23 910 (16.7%)
3	15 072 (19.6%)	28 172 (19.7%)
4	14 498 (18.8%)	27 341 (19.1%)
5	10 223 (13.3%)	19 675 (13.8%)
Missing data	10 604 (13.8%)	18 028 (12.6%)
BMI (kg/m^2^; mean (s.d.)	28.6 (7.6)	27.4 (6.4)
BMI categorized		
<25 kg/m^2^	25 739 (33.4%)	52 666 (36.8%)
25–29.99 kg/m^2^	15 065 (19.6%)	29 682 (20.8%)
≥30 kg/m^2^	24 139 (31.4%)	37 585 (26.3%)
Missing or implausible data	12 035 (15.6%)	23 144 (16.2%)
Smoking status		
Non-smokers	56 105 (72.9%)	101 370 (70.8%)
Smokers	17 383 (22.6%)	32 778 (22.9%)
Missing or implausible data	3490 (4.5%)	8929 (6.2%)
Medical conditions at baseline		
Diabetes	1694 (2.2%)	1449 (1.0%)
Hypertension	2325 (3.0%)	2805 (2.0%)
Hypothyroidism	2987 (3.9%)	3228 (2.3%)
Impaired glucose regulation†	446 (0.6%)	368 (0.3%)
Ethnicity		
Caucasian-European	36 670 (47.6%)	58 182 (40.7%)
Black-Afro-Caribbean	1692 (2.2%)	3222 (2.3%)
South Asians	3929 (5.1%)	4266 (3.0%)
Others including Chinese, Middle-Eastern	1013 (1.3%)	1781 (1.2%)
Mixed-race	715 (0.9%)	1008 (0.7%)
Missing (no codes found in Medical or AHD file)	32 959 (42.8%)	74 618 (52.2%)

*Townsend score – presented as quintiles with 1 least deprived and 5 most deprived. ^†^(Includes impaired fasting glucose and impaired glucose tolerance).

The clinical features of PCOS and medications used in the study cohort are summarized in Table 2. Of the 76 978 women with PCOS, 22 307 (29.0%) were defined using PCOS Read Code while the remainder were defined using PCO Read Code (see Supplementary Table E1).

**Table 2:** PCOS diagnostic features and use of medication. Data are presented as *n* (%)

Characteristics	With PCOS (*n* = 76 978)	Without PCOS (*n* = 143 077)
PCOS features at baseline		
Anovulation	20 106 (26.1)	10 463 (7.3)
Any clinical androgen excess feature	21 974 (28.6)	23 231 (16.3)
Acne	14 589 (19.0)	19 371 (13.5)
Alopecia	2749 (3.6)	3820 (2.7)
Hirsutism	7748 (10.1)	1269 (0.9)
Polycystic ovaries	58 099 (75.5)	0 (0)
Medications		
Oral Contraceptive Pill (OCP) including an anti-androgenic progestin component*	22 798 (29.6)	17 005 (11.9)
Cyproterone	17 346 (22.5)	9381 (6.6)
Drospirenone	9685 (12.6)	10 280 (7.2)
Other antiandrogen	192 (0.2)	14 (0.0)
Metformin	18 571 (24.1)	2925 (2.0)

*Use of either cyproterone acetate or drospirenone. Since some patients were prescribed both cyproterone and drospirenone during the period covered, the number of OCP users is slightly smaller than the arithmetic sum of the numbers of cyproterone and drospirenone users.

### Primary analysis

There were 298 incident cases of OSA among 76 978 women with PCOS and 222 among 143 077 women without PCOS. Incidence of OSA was significantly higher in women with PCOS vs the matched controls (8.1 vs 3.3 per 10 000 person-years of follow-up, *P* < 0.0001) (Table 3). Women with PCOS had an increased hazard of developing OSA when compared to their matched controls (HR = 2.46, 95% CI: 2.07–2.93, *P* < 0.001). Women with PCOS remained at increased risk of developing OSA compared to women without PCOS following adjustment for age, Townsend score, BMI, hypothyroidism at baseline, baseline and incident diabetes/IGR (adjusted HR = 2.26, 95% CI: 1.89 to 2.69, *P* < 0.001) (Table 3).

**Table 3:** Risk of women with PCOS developing OSA compared to women without PCOS.

	Primary analysis		Sensitivity analysis (incident cases)		Sensitivity analysis (PCOS specific Read Codes only)	
	Exposed	Unexposed	Exposed	Unexposed	Exposed	Unexposed
Total number of patients	76 978	143 077	23 349	43 106	24 603	44 991
Incident OSA *n* (%)	298 (0.39)	222 (0.16)	86 (0.37)	60 (0.14)	115 (0.47)	63 (0.14)
Person years	368 203	680 011	112 290	193 646	107 959	187 801
Incidence rates per 10 000 person years	8.1	3.3	7.7	3.1	10.7	3.4
Hazard ratio (95% CI)	2.46 (2.07–2.93)		2.44 (1.76–3.40)		3.11 (2.29–4.23)	
*P*-value	<0.001		<0.001		<0.001	
Adjusted hazard ratio (95% CI)*	2.26 (1.89–2.69)		2.20 (1.58–3.06)		2.93 (2.15–3.99)	
*P*-value	<0.001		<0.001		<0.001	
Adjusted hazard ratio (95% CI)^†^	2.23 (1.87–2.66)		2.14 (1.53–2.99)		2.87 (2.10–3.92)	
*P*-value	<0.001		<0.001		<0.001	

*Adjusted for age, Townsend score, BMI, diabetes or impaired glucose regulation at baseline and hypothyroidism at baseline. ^†^Adjusted for age, Townsend score, BMI, diabetes or impaired glucose regulation up to end of follow-up and hypothyroidism at baseline.

In addition to PCOS, we identified older age, increasing BMI and the presence of hypothyroidism at baseline as independent predictors of incident OSA (see Supplementary Table E2).

### Sensitivity analysis

Limiting the analysis to incident cases of PCOS (adjusted HR = 2.20, 95% CI: 1.58–3.06, *P* < 0.001) or PCOS Read Codes only (adjusted HR = 2.93, 95% CI: 2.15–3.99, *P* < 0.001) resulted in similar point estimates to those found in the analysis of the whole study population (Table 3). Adding ethnicity as an additional covariate did not alter our findings (adjusted HR 2.21, 95% CI: 1.85–2.64, *P* < 0.001).

### Analysis to assess if risk of OSA among women with PCOS is independent of BMI

**Figure 2:**
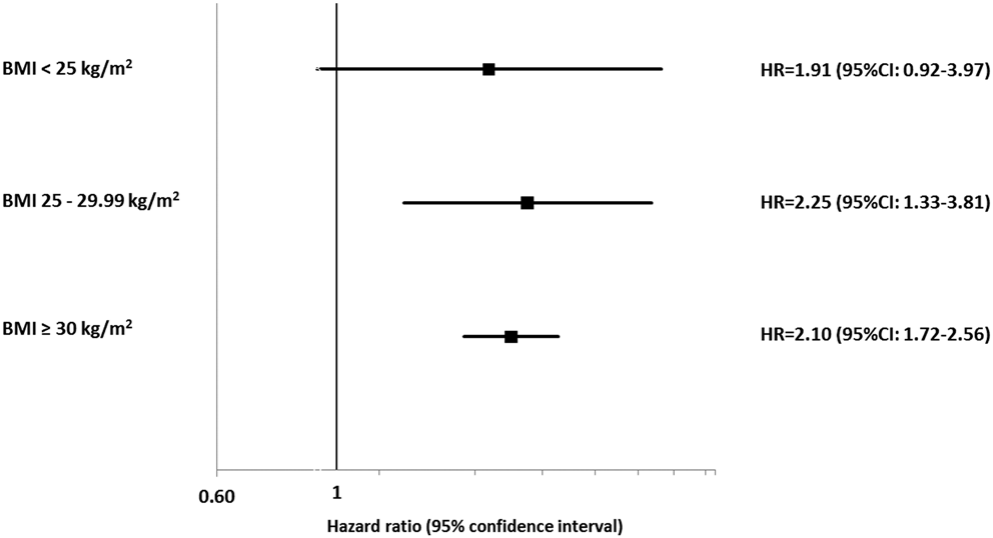
Impact of BMI on the hazard of obstructive sleep apnoea (OSA). Women in both PCOS and control cohorts were subcategorized into lean, overweight and obese groups. The hazard of developing OSA among PCOS cohort in comparison to control cohort was assessed for each BMI sub groups.

Increasing BMI was associated with increasing OSA incidence in women with and without PCOS, but the HRs were greater in women with PCOS vs control in all BMI categories (see Fig. 2 and Supplementary Table E2). When compared to women without PCOS, women with PCOS showed a higher risk (adjusted HR (95% CI)) of developing OSA in all three BMI (kg/m^2^) categories (BMI <25: 1.91 (0.92–3.97), *P* = 0.081; BMI 25–29.99: 2.25 (1.33–3.81), *P* = 0.003; BMI ≥30: 2.10 (1.72–2.56), *P* < 0.001) (see Fig. 2 and Supplementary Table E3).

### Analysis to assess the role of factors other than BMI in OSA among PCOS women

In addition to older age, increasing BMI and presence of hypothyroidism, we identified anovulation (adjusted HR (95% CI) 1.33 (1.05–1.69), *P* = 0.02), hirsutism (1.37 (1.05–1.80), *P* = 0.02) and treatment with metformin (1.69, 1.28–2.22, *P* < 0.001) as predictors of incident OSA (Table 4).

**Table 4:** Predictors of OSA in women with PCOS (*n* = 76 978).

Covariates	Hazard ratio	95% confidence interval	*P* value
Age	1.06	(1.04–1.07)	**<0.001**
Townsend			
1	1.00	(Reference level)	
2	1.26	(0.83–1.89)	0.27
3	1.41	(0.95–2.08)	0.08
4	1.58	(1.07–2.33)	**0.02**
5	1.67	(1.10–2.53**)**	**0.02**
Missing	1.23	(0.77–1.97)	0.38
BMI category			
<25 kg/m^2^	1.00	(Reference level)	
25–29.99 kg/m^2^	3.01	(1.60–5.69)	**0.001**
≥30 kg/m^2^	12.50	(7.24–21.58)	**<0.001**
Missing	3.64	(1.91–6.95**)**	**<0.001**
Diabetes or IGR*	0.72	(0.44–1.18)	0.19
Hypothyroidism	1.76	(1.20–2.58)	**0.004**
Anovulation	1.33	(1.05–1.69)	**0.02**
Androgen excess features			
Hirsutism	1.37	(1.05–1.80)	**0.02**
Acne	0.85	(0.61–1.19)	0.35
Alopecia	0.97	(0.64–1.46)	0.89
Metformin	1.69	(1.28–2.22)	**<0.001**
Antiandrogen drug	0.77	(0.57–1.05)	0.10

*Impaired glucose response (includes impaired fasting glucose and impaired glucose tolerance). Values in boldface indicate statistical significance.

## Discussion

This is the first large longitudinal study assessing the temporal relationship between PCOS and OSA in a European population and that accounted for the role of obesity and adjusted for a large number of potential confounders. We found that women with PCOS were at increased risk of developing OSA compared women without PCOS regardless of the severity of obesity and independent of a wide range of potential confounders. We also found that hirsutism and chronic anovulation, clinical features of androgen excess, which is closely linked to insulin resistance in the context of PCOS, were independent predictors of OSA development in women with PCOS.

A recent study from Taiwan also found an increase OSA incidence in women with PCOS, but was of much smaller sample size (*n* = 9190 vs 220 055) and did not match for BMI; in addition, the adjustment for confounders was limited and did not explore the possible underlying mechanisms (22).

The prevalence of OSA in women with PCOS has been investigated in several small-scale cross-sectional studies (9). Two recent meta-analyses showed an estimated OSA prevalence (based on polysomnography or portable level II devices) of 36.1% (95% CI: 22.4–51.0%) and 32% (95% CI: 13–55%) in women with PCOS (23, 24). However, the prevalence of OSA in women with PCOS varied widely from 0 to 69% (9). This big variation in OSA prevalence between studies is attributed to multiple factors, including the application of different AHI cut-offs to diagnose OSA, small sample sizes, variation in obesity prevalence and potential selection bias due to recruitment from specialized clinics, raising the possibility that the results many not be generalizable (9). Our current study addresses many of these gaps by conducting a population-based longitudinal study of a large sample size that included patients with normal, overweight and obese BMI categories. The impact of PCOS on risk of OSA was stronger in overweight than in obese women; however, this needs to be interpreted with caution given the confidence intervals of effect sizes overlap.

The cross-sectional studies that showed low prevalence rates of OSA in women with PCOS were mainly carried out in leaner cohorts, hence suggesting that the high prevalence of OSA in women with PCOS was only applicable to women with obesity. Whether women with PCOS were truly at increased risk of OSA compared to women with similar obesity but without PCOS was unclear (9, 23, 24). Our study addressed this limitation of earlier studies by including a control group of women matched for BMI amongst other important covariates, and our results show clearly that women with PCOS were at increased risk OSA compared to women without PCOS. In addition, we have shown that this increased risk of OSA in women with PCOS was evident irrespective of whether patients were normal weight, overweight or have obesity. However, the observed relationships were stronger in women who were overweight or had obesity.

Several mechanisms might explain the link between PCOS and OSA and explain the findings of this study (9, 25). Androgen excess is a cardinal feature of PCOS and androgens are implicated in the pathogenesis of OSA as they affect upper airway stability and ventilatory drive (9). OSA is consistently more common in men than women (26, 27), which may also point to androgens as a contributing factor. Our data support this notion as a documented diagnosis of hirsutism, a clinical manifestation of androgen excess, was an independent predictor of incident OSA. In addition to androgen excess, oxidative stress and low progesterone might play a role in the observed relationship between PCOS and OSA (25). Oxidative stress can contribute to the pathogenesis of OSA by causing dysfunction in the carotid body chemoreceptors resulting in ventilatory instability (28). Progesterone is a respiratory stimulant and can result in lowering upper respiratory resistance (29). Furthermore, OSA is associated with lower progesterone in women (30) and low progesterone affects the ventilatory drive (9, 31, 32). Our results also support this potential mechanism as anovulation predicted incident OSA. Insulin resistance, a feature of OSA and a predictor of incident apnoea can also contribute to the observed relationships in this study (33, 34).

OSA is associated with multiple comorbidities including road traffic accidents, hypertension, T2D, CVD, and mortality, which are similar to PCOS comorbidities (except the road traffic accidents). However, whether OSA increases the risk of these comorbidities in women with PCOS is largely unknown. A recent systematic review and meta-analysis showed that women with both PCOS and OSA are more likely to have hirsutism, hypertension, insulin resistance, hyperlipidaemia, and dysglycaemia than women with PCOS only (24). However, these findings were based on small-scale cross-sectional studies with a high risk of bias (9). Hence, longitudinal and interventional studies to assess the temporal relationship and causation are needed.

The findings of our study need to be interpreted within the context of the limitations. We carried out an observational cohort study using data from primary care and hence we did not have access to the results of the polysomnography, polygraphy or oximetry such as the AHI, oxygen desaturation index and other hypoxemic measures. In addition, since we and other studies (14, 17) have observed a much lower prevalence of PCOS and OSA in electronic medical records, it is possible that many patients with PCOS and OSA remained undiagnosed. This may be due lack of diagnosis or incomplete recording of PCOS and OSA diagnosis in primary care setting. It is possible that patients with PCOS who come to the attention of the treating clinician represented the more severe phenotype which may have resulted in an overestimate of the effect size. This may limit the generalizability of our findings to patients with milder form of disease. On the other hand, the unexposed population may have patients with undiagnosed PCOS which could potentially lead to an underestimation of the effect size.

Nevertheless, our study has several strengths including the large sample size, the longitudinal nature of the study, the matched controlled design and the adjustment for a wide range of confounders including BMI. In addition, this was a population-based study rather than data from tertiary centres and hence the findings are much more generalizable. The longitudinal design of the study also allowed us to assess the predictors of OSA in women with PCOS.

We conclude that women with PCOS are at increased risk of developing OSA compared to women without PCOS independent of obesity and other potential cofounders. This increased risk was present in patients with normal weight, overweight and obesity. Clinicians treating women with PCOS should have a low threshold to test for OSA. In addition, our findings suggest that clinical features of hyperandrogenism and low progesterone may contribute to the increased risk of OSA in women with PCOS. Whether OSA worsens the long-term sequelae of PCOS and whether CPAP treatment has favourable impacts on women with PCOS and OSA remains to be examined.

## Supplementary Material

Supplementary Fig. E1

Detailed methodology

Panel E1. Options for methodical selection of Read codes

Table E1: Read codes used to identify polycystic ovary syndrome

E2 Table: Regression model estimates for hazard of women with PCOS to develop OSA compared to women without PCOS (n=220,055)

E3 Table: Subgroup analysis for hazard of women with PCOS to develop OSA compared to women without PCOS stratified by BMI category

## Declaration of interest

The authors declare that there is no conflict of interest that could be perceived as prejudicing the impartiality of this study.

## Funding

A A T is a clinician scientist supported by the National Institute for Health Research (NIHR) in the UK (CS-2013-13-029); A C is an NIHR Senior Investigator. The views expressed in this publication are those of the authors and not necessarily those of the National Health Service, the NIHR or the Department of Health. W A is supported by the Wellcome Trust (Investigator Award in Science, 209492/Z/17/Z).
