# Supplementary material for: Increased risk of obstructive sleep apnoea in women with polycystic ovary syndrome: a population-based cohort study
Source: Eur J Endocrinol. 2019 Feb 13;180(4):265–72. doi: 10.1530/EJE-18-0693 (PMC6410684; doi:10.1530/EJE-18-0693)
Supplement: Supplementary Fig. E1 [file supplementary_figure_1.pdf]

Visual representation of study timeline and selection process (Identification of the exposed group (individuals with PCOS/PCO Read codes) and their controls (without PCOS/PCO) matched 1:2 by age at index date, BMI and general practice)

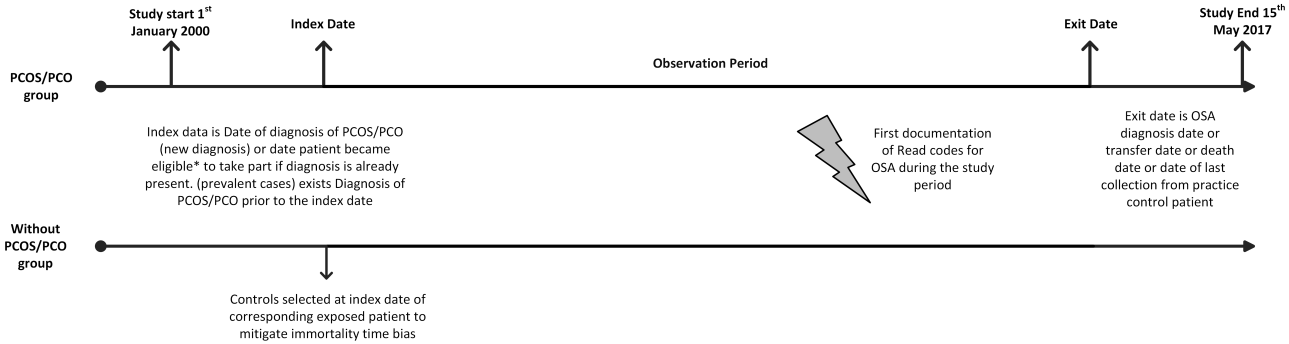

* To ensure good quality data, a patient is eligible to take part one year after the latest of the following dates: 1) registration with the general practice (registration date); 2) introduction of VISION Electronic Medical Record (VISION date); and 3) Acceptable Mortality Recording (AMR) date. AMR is an indicator when practices started to record information consistently and in a timely manner[1,2]**.** One year latent period is applied to ensure there was sufficient time to record all important covariates.

1. Maguire A, Blak BT, Thompson M. The importance of defining periods of complete mortality reporting for research using automated data from primary care. *Pharmacoepidemiol Drug Saf* 2009; **18**(1): 76-83.
2. Horsfall L, Walters K, Petersen I. Identifying periods of acceptable computer usage in primary care research databases. *Pharmacoepidemiol Drug Saf* 2013; **22**(1): 64-9.

Figure E.1: Visual presentation of study selection process
