# Supplementary material for: Increased risk of obstructive sleep apnoea in women with polycystic ovary syndrome: a population-based cohort study
Source: Eur J Endocrinol. 2019 Feb 13;180(4):265–72. doi: 10.1530/EJE-18-0693 (PMC6410684; doi:10.1530/EJE-18-0693)
Supplement: Detailed methodology [file supplementary_data_1.pdf]

Detailed methodology

Study design and setting

We conducted a matched-controlled retrospective cohort study using data from UK general practices contributing to The Health Improvement Network (THIN) electronic database, that was used previously in multiple studies of similar design to the current study [1–3]. It contains longitudinal records of clinical, laboratory and therapeutic information of each encounter with the patients registered with these practices using a hierarchical clinical 'Read Code' system [4] THIN covers around 6% of UK population (3.7 million) from 763 practices representatively scattered around the UK [5] The generalizability of THIN has been validated for pharmaco-epidemiological research [6] and THIN has been used previously in studies examining PCOS [7] and OSA [8] .

Study population

The open cohort extended from the 1st January 2000 (study start date) to the 15th May 2017 (study end date). All individuals were required to be registered at their practice at least for a year before entry into the study in order to ascertain documentation of concomitant diseases. Their practice was also required to have been using their computer system (Vision) for at least one year prior to the index date (the date at which each participant joined the cohort) and have an acceptable mortality reporting in order to ensure good data quality [9].

All women who fulfilled the above-mentioned criteria and who were aged 18-50 years at the index date and had a documentation of PCOS at any time during the study period were included in the exposed group. Patients with any documentation of OSA prior to the index date were excluded. Women without documented PCOS at any time during the study period were included in the unexposed (control) arm. The index date was defined as the date of first documentation of PCOS for newly diagnosed cases and from the date patient became eligible

if the first documentation of PCOS was prior to the eligibility date (for existing cases) (see Figure E1 in the online data supplement.).

Each exposed patient was randomly matched to 2 unexposed patients (1:2 ratio) for general practice, age at index date (± one year) and BMI (± 2 kg/m^2). Matching variables were chosen based on their strong association with PCOS and OSA [8] [10] When more than 2 participants in the unexposed cohort were available to be matched with a participant in the exposed cohort, two were randomly selected.

To minimize the immortal time bias, each randomly matched eligible unexposed patient was assigned the same index date as their corresponding exposed patient [11]. Follow up end date (exit date) was determined from the earliest occurrence of: the first documentation of OSA, transfer to another practice, death or study-end.

Selection of Read Codes and PCOS definition

Read Codes to define PCOS, OSA and covariates (see Tables E1 to E17 in the online data supplement.) were compiled using a methodical Read Code search strategy (see Panel E1 in the online data supplement.) [12] including codes used in other studies [7] [8]. Since there is a possibility of misclassification between PCOS and Polycystic ovaries (PCO) due to the resemblance of codes during data entry, they have been combined in prevalence studies using general practice electronic databases [7].

Grouping of quantitative variables

BMI (in kg/m^2) was categorized into normal (<25), overweight (25 to 29.99) and obese (≥30) [13]. Impaired glucose regulation (IGR) included impaired fasting glucose (IFG; fasting plasma glucose 6.1-6.9 mmol/L) and impaired glucose tolerance (IGT; plasma glucose 7.8-11.1 mmol/L measured 120min after ingestion of 75g glucose in the oral glucose tolerance test).

Statistical analysis

Potential confounders and covariates were chosen based on biological plausibility and links to the exposure and outcome of interest (PCOS and OSA respectively). These included: age, Townsend social deprivation index [8], BMI, smoking status, diabetes mellitus, impaired glucose regulation [14] hypertension [15], hypothyroidism, antiandrogen medication, and metformin. Baseline characteristics were summarized considering the distribution of data and without statistical testing to comply with the statistical guidelines [16] [17] . The critical value for statistical significance was set at 5% and therefore 95% confidence intervals were used in the population estimate of hazard ratios. Further analysis included calculating the number and percentage of incident cases, person years, and incidence rates. Unadjusted and adjusted hazard ratios were estimated using Cox regression models. The initial adjusted model included age, Townsend score, BMI, diabetes or IGR and hypothyroidism at baseline as covariates. Sensitivity analysis was carried out to assess selection bias due to case definition (PCOS and PCO vs PCOS only) and survival bias due to inclusion of prevalent cases (who had the documentation of PCOS prior to becoming eligible for the study) [18]. Another model confined to PCOS cases (excluding controls) was used to assess the association of PCOS with OSA while considering PCOS phenotypes and antiandrogen medication as covariates. STATA MP version 14.2 was used for data cleaning and analysis [19] 21.

References

1. Kumarendran B, O'Reilly MW, Manolopoulos KN, Toulis KA, Gokhale KM, Sitch AJ, Wijeyaratne CN, Coomarasamy A, Arlt W, Nirantharakumar K. Polycystic ovary syndrome, androgen excess, and the risk of nonalcoholic fatty liver disease in women: A longitudinal study based on a United Kingdom primary care database. PLoS Med. 2018;15(3).

2. Caleyachetty R, Thomas GN, Toulis KA, Mohammed N, Gokhale KM, Balachandran K, Nirantharakumar K. Metabolically Healthy Obese and Incident Cardiovascular Disease Events Among 3.5 Million Men and Women. J Am Coll Cardiol [Internet]. 2017 [cited 2017 Sep 12];70(12). Available from: http://www.onlinejacc.org/content/70/12/1429?sso=1&sso_redirect_count=1&access_token=

3. Toulis KA, Willis BH, Marshall T, Gokhale K, Frcp SG, Thomas GN. All-cause mortality in patients with diabetes under treatment with dapagliflozin: a population-based, open-cohort study in THIN database. J Clin Endocrinol Metab [Internet]. 2017; Available from: https://academic.oup.com/jcem/article/doi/10.1210/jc.2016-3446/3038070/Allcause-mortality-in-patients-with-diabetes-under

4. Booth N. What are the Read Codes? [Internet]. Vol. 11, Health libraries review. 1994. p. 177–82. Available from: http://www.ncbi.nlm.nih.gov/pubmed/10139676

5. INPS. About us - The Health Improvement Network (THIN) [Internet]. 2017 [cited 2017 Jun 6]. Available from: http://www.inps.co.uk/vision/thin/about-us

6. Lewis JD, Schinnar R, Bilker WB, Wang X, Strom BL. Validation studies of the health improvement network (THIN) database for pharmacoepidemiology research. Pharmacoepidemiol Drug Saf [Internet]. 2007 Apr [cited 2016 Jul 1];16(4):393–401. Available from: http://www.ncbi.nlm.nih.gov/pubmed/17066486

7. Ding T, Baio G, Hardiman PJ, Petersen I, Sammon C. Diagnosis and management of polycystic ovary syndrome in the UK): a retrospective cohort study. 2016;5–8. Available from: http://dx.doi.org/

8. Wall H, Smith C, Hubbard R. Body mass index and obstructive sleep apnoea in the UK: a cross-sectional study of the over-50s. Prim Care Respir J [Internet]. 2012 Dec 29 [cited 2015 Nov 22];21(4):371–6. Available from: http://www.nature.com/articles/pcrj201253

9. Maguire A, Blak BT, Thompson M. The importance of defining periods of complete mortality reporting for research using automated data from primary care. Pharmacoepidemiol Drug Saf [Internet]. 2009 Jan [cited 2015 Nov 22];18(1):76–83. Available from: http://www.ncbi.nlm.nih.gov/pubmed/19065600

10. Lim SS, Davies MJ, Norman RJ, Moran LJ. Overweight, obesity and central obesity in women with polycystic ovary syndrome: a systematic review and meta-analysis. Hum Reprod Update [Internet]. 2012;18(6):618–37. Available from: http://humupd.oxfordjournals.org/cgi/doi/10.1093/humupd/dms030

11. Toulis KA, Willis BH, Marshall T, Kumarendran B, Gokhale K, Ghosh S, Thomas GN, Cheng KK, Narendran P, Hanif W, Nirantharakumar K. All-Cause Mortality in Patients With Diabetes Under Treatment With Dapagliflozin: A Population-Based, Open-Cohort Study in The Health Improvement Network Database. J Clin Endocrinol Metab [Internet]. 2017 May 1 [cited 2018 Mar 21];102(5):1719–25. Available from: http://www.ncbi.nlm.nih.gov/pubmed/28323967

12. Davé S, Petersen I. Creating medical and drug code lists to identify cases in primary care databases. Pharmacoepidemiol Drug Saf [Internet]. 2009 Aug [cited 2017 May 8];18(8):704–7. Available from: http://doi.wiley.com/10.1002/pds.1770

13. Physical status: the use and interpretation of anthropometry. Report of a WHO Expert Committee. World Health Organ Tech Rep Ser [Internet]. 1995 [cited 2018 Feb 14];854:1–452. Available from: http://www.ncbi.nlm.nih.gov/pubmed/8594834

14. Moran LJ, Misso ML, Wild RA, Norman RJ. Impaired glucose tolerance, type 2 diabetes and metabolic syndrome in polycystic ovary syndrome: a systematic review and meta-analysis. Hum Reprod Update [Internet]. 2010;16(4):347–63. Available from: http://humupd.oxfordjournals.org/cgi/doi/10.1093/humupd/dmq001

15. McEvoy RD. Obstructive sleep apnoea and hypertension: the ESADA study. Eur Respir J [Internet]. 2014 Oct 1 [cited 2017 Sep 24];44(4):835–8. Available from:

http://www.ncbi.nlm.nih.gov/pubmed/25271222

16. Benchimol EI, Smeeth L, Guttmann A, Harron K, Moher D, Petersen I, Sørensen HT, von Elm E, Langan SM. The REporting of studies Conducted using Observational Routinely-collected health Data (RECORD) Statement. PLoS Med. 2015;12(10):1–22.

17. Bland JM, Altman DG. Multiple significance tests: the Bonferroni method. BMJ [Internet]. 1995 [cited 2017 May 24];310(6973). Available from: http://www.bmj.com/content/310/6973/170.long

18. Matok I, Azoulay L, Yin H, Suissa S. Immortal time bias in observational studies of drug effects in pregnancy. Birth Defects Res A Clin Mol Teratol [Internet]. 2014 Sep [cited 2015 Nov 11];100(9):658–62. Available from: http://www.ncbi.nlm.nih.gov/pubmed/24985740

19. StataCorp. Stata Statistical Software: Release 14 [Internet]. 2015. TX: StataCorp LP.: College Station; 2015 [cited 2017 May 26]. Available from: http://www.stata.com/statamp/
