# Supplementary material for: Increased risk of obstructive sleep apnoea in women with polycystic ovary syndrome: a population-based cohort study
Source: Eur J Endocrinol. 2019 Feb 13;180(4):265–72. doi: 10.1530/EJE-18-0693 (PMC6410684; doi:10.1530/EJE-18-0693)
Supplement: Panel E1. Options for methodical selection of Read codes [file supplementary_data_2.pdf]

Panel E1. Options for methodical selection of Read codes

**Options for methodical selection of Read codes**
• System based grouping<br>• Use of flags in prioritizing codes into difference hierarchy based on relevance<br>• Read codes cited in previous studies<br>• Read codes listed in Clinical codes.org<br>• Read codes corresponding to Quality Outcome Framework (QoF)<br>• Applying search for key words<br>• Discussion within the team (First attempt)<br>• Discussion with GPs
