# Supplementary material for: Increased risk of obstructive sleep apnoea in women with polycystic ovary syndrome: a population-based cohort study
Source: Eur J Endocrinol. 2019 Feb 13;180(4):265–72. doi: 10.1530/EJE-18-0693 (PMC6410684; doi:10.1530/EJE-18-0693)
Supplement: Table E1: Read codes used to identify polycystic ovary syndrome [file supplementary_data_3.pdf]

Table E1: Read codes used to identify polycystic ovary syndrome

Read Code	Description
**Polycystic ovary syndrome**	
C164.11	Iso-sexual virilisation
C164.12	Stein - Leventhal syndrome
C165.00	Polycystic ovarian syndrome
7E25300	Endoscopic drilling of ovary
**Polycystic ovaries**	
C164.00	Polycystic ovaries
C164.13	Multi-cystic ovaries
**Sleep apnoea**	
Fy03.00	Sleep apnoea
Fy03.11	Obstructive sleep apnoea
Fy04.11	Ondine's curse
H5B..00	Sleep apnoea
H5B0.00	Obstructive sleep apnoea
R005100	[D]Insomnia with sleep apnoea
R005300	[D]Hypersomnia with sleep apnoea
R005311	[D]Sleep apnoea syndrome
R005312	[D]Syndrome sleep apnoea
R060400	[D]Apnoea
