# Supplementary material for: Increased risk of obstructive sleep apnoea in women with polycystic ovary syndrome: a population-based cohort study
Source: Eur J Endocrinol. 2019 Feb 13;180(4):265–72. doi: 10.1530/EJE-18-0693 (PMC6410684; doi:10.1530/EJE-18-0693)
Supplement: E2 Table: Regression model estimates for hazard of women with PCOS to develop OSA compared to women without PCOS (n=220,055) [file supplementary_data_4.pdf]

E2 Table: Regression model estimates for hazard of women with PCOS to develop OSA compared to women without PCOS (n=220,055)

Covariate	Hazard ratio	95% Confidence interval		P-value
**PCOS**	**2.26**	**(1.89**	**2.69)**	**<0.001**
**Age**	**1.05**	**(1.04**	**1.06)**	**<0.001**
Townsend score#				
1	1.00	(Reference category)		
2	1.22	(0.89	1.66)	0.220
3	1.29	(0.95	1.73)	0.100
**4**	**1.51**	**(1.12**	**2.03)**	**0.007**
**5**	**1.76**	**(1.30**	**2.40)**	**<0.001**
**Missing or implausible data**	**1.43**	**(1.01**	**2.01)**	**0.044**
BMI Category (kg/m^2)				
<25	1.00	(Reference level)		
**25-29.99**	**2.94**	**(1.88**	**4.60)**	**<0.001**
**>30**	**13.69**	**(9.37**	**20.0)**	**<0.001**
**Missing or implausible data**	**2.34**	**(1.41**	**3.87)**	**0.001**
Diabetes or Impaired glucose regulation*	1.26	(0.87	1.81)	0.223
**Hypothyroidism**	**2.02**	**(1.50**	**2.71)**	**<0.001**

Townsend score – presented as quintiles with 1 least deprived and 5 most deprived

* (includes Impaired Fasting Glucose and Impaired Glucose Tolerance)
