# Supplementary material for: Increased risk of obstructive sleep apnoea in women with polycystic ovary syndrome: a population-based cohort study
Source: Eur J Endocrinol. 2019 Feb 13;180(4):265–72. doi: 10.1530/EJE-18-0693 (PMC6410684; doi:10.1530/EJE-18-0693)
Supplement: E3 Table: Subgroup analysis for hazard of women with PCOS to develop OSA compared to women without PCOS stratified by BMI category [file supplementary_data_5.pdf]

E3 Table: Subgroup analysis for hazard of women with PCOS to develop OSA compared to women without PCOS stratified by BMI category

	**BMI <25kg/m2**		**25-29.99 Kg/m2**		**≥30Kg/m2**	
	**PCOS (Exposed)**	**Controls (Unexposed)**	**PCOS (Exposed)**	**Controls (Unexposed)**	**PCOS (Exposed)**	**Controls (Unexposed)**
Total number of participants	25,725	52,651	15,035	29,656	23,912	37,409
Person years	117,924	243,863	72,979	144,545	117,242	186,177
Incident OSA n (%)	14 (0.05)	15 (0.03)	30 (0.20)	26 (0.09)	227 (0.94)	176 (0.47)
Incidence rates per 10,000 person years	1.19	0.62	4.1	1.80	19.4	9.45
Hazard Ratio (95% CI)	1.92 (0.92 - 3.97)		2.25 (1.33 - 3.81)		2.05 (1.68 - 2.49)	
p-value	0.080		0.002		<0.001	
Adjusted Hazard Ratio (95% CI)*	1.91 (0.92 - 3.97)		2.25 (1.33 - 3.81)		2.10 (1.72 - 2.56)	
p-value	0.081		0.003		<0.001	

* Adjusted for age, Townsend score, BMI, diabetes or impaired glucose regulation and hypothyroidism at baseline
